# Post-Translational Modifications in Tumor-Associated Antigens as a Platform for Novel Immuno-Oncology Therapies

**DOI:** 10.3390/cancers15010138

**Published:** 2022-12-26

**Authors:** Anurag Kumar Srivastava, Giorgia Guadagnin, Paola Cappello, Francesco Novelli

**Affiliations:** 1 Department of Molecular Biotechnology and Health Sciences, University of Turin, 10126 Turin, Italy; 2 Center for Experimentals and Medical Research, Azienda Ospedaliera Universitaria, Città della Salute e della Scienza di Torino, 10126 Turin, Italy

**Keywords:** tumor-associated antigens, cancer immunotherapy, post-translational modifications, acetylation, citrullination, phosphorylation, glycosylation

## Abstract

**Simple Summary:**

Tumor-associated antigens (TAAs) are antigens present in tumor cells, but are also expressed in normal cells. However, TAAs are aberrantly expressed by tumor cells, and can elicit multiple specific immune responses. One key feature of TAAs is the presence of post-translational modifications often absent in normal proteins. This article offers an overview of the role of post-translational modifications in TAAs in eliciting a specific immune response, which makes them targets for immuno-oncology therapy. Both preclinical and clinical studies will be discussed.

**Abstract:**

Post-translational modifications (PTMs) are generated by adding small chemical groups to amino acid residues after the translation of proteins. Many PTMs have been reported to correlate with tumor progression, growth, and survival by modifying the normal functions of the protein in tumor cells. PTMs can also elicit humoral and cellular immune responses, making them attractive targets for cancer immunotherapy. This review will discuss how the acetylation, citrullination, and phosphorylation of proteins expressed by tumor cells render the corresponding tumor-associated antigen more antigenic and affect the immune response in multiple cancers. In addition, the role of glycosylated protein mucins in anti-cancer immunotherapy will be considered. Mucin peptides in combination with stimulating adjuvants have, in fact, been utilized to produce anti-tumor antibodies and vaccines. Finally, we will also outline the results of the clinical trial exploiting glycosylated-MUC1 as a vaccine in different cancers. Overall, PTMs in TAAs could be considered in future therapies to result in lasting anti-tumor responses.

## 1. Introduction

Post-translation modifications (PTMs) add small chemical moieties or chemical modifications at individual amino acids in translated proteins. PTMs regulate protein stability, folding, function, and their interaction with other biomolecules [1]. The most characterized PTMs are phosphorylation, acetylation, glycosylation, citrullination, and ubiquitination [2]. PTMs are frequently involved in many diseases beside cancer. Highly phosphorylated tau protein has been associated with neurodegenerative diseases, including Alzheimer’s disease [3]. In rheumatoid arthritis, protein hypercitrullination is a hallmark of the disease, and autoantibodies to hyper-citrullinated proteins are typically detected in the patient’s synovial fluid [4]. In type 2 diabetes (T2D), an increase in global glycosylation levels leads to impaired release of secreted proteins from adipose tissue and also induces insulin resistance [5,6].

Cancer cell transformation profoundly changes gene assets, and consequently protein expression and activation. Tumor antigens are classified into: i) tumor specific antigens (TSAs) and ii) tumor-associated antigens (TAAs). TSAs are proteins specifically expressed by tumors but not by normal cells. TSAs can be classified into wild type TSAs and mutated TSAs or “neoantigens” [7]. The latter are proteins with individual specificity and emerge from somatic mutations in the tumor genome [8]. As each tumor displays an individual heterogeneity and mutational burden, neoantigens can be defined as truly tumor specific. Wild-type TSAs have been identified as human leukocyte antigen (HLA)-eluted peptides with wild-type sequences (compared with the relevant germline sequence) that, nonetheless, have tumor specific presentation, not represented on benign/normal tissues, and to which the immune system has not been previously exposed [7]. TSA were also identified and characterized by using high-throughput multi-omics analyses, including next-generation sequencing or tandem mass spectrometry (MS/MS) [7].

Self-proteins expressed in both tumor and non-malignant cells but aberrantly present in tumor cells in terms of amount, chemical features, location or time, are defined as TAAs and can elicit multiple specific immune responses [9]. TAAs were identified by using serological proteome analysis (SERPA) or serological analysis of recombinant cDNA expression libraries (SEREX) [10]. Between these two approaches to identify TAAs, SERPA, which specifically identifies the different isoforms of tumor proteins, is the more appropriate way to also identify the autoantibody response to PTM [11]. A key example of a TAA identified by SERPA is alpha-enolase (ENO1), which is overexpressed, acetylated, or phosphorylated in pancreatic cancer [12,13,14,15,16]. Other examples of TAAs that have been identified thanks to the analysis of the immune response elicited by them in cancer patients include acetylated and phosphorylated p53, phosphorylated insulin receptor substrate 2 (IRS2), cell division cycle 25b (CDC25b), citrullinated vimentin (Vim), and glycosylated mucin (MUC) protein [17,18,19,20,21].

The choice of TSAs or TAAs is essential in any strategy aiming to unleash the antitumor T cell response with vaccine approaches [22]. Therapeutic cancer vaccines allow peptides derived from tumor proteins (TSAs or TAAs previously identified) to be presented by HLA molecules in order to activate the immune system to recognize and kill the established tumors expressing those proteins. These vaccines typically involve exogenous administration of selected tumor antigens combined with adjuvants that activate dendritic cells (DCs) as antigen presenting cells, or even DCs themselves previously loaded with the tumor antigen [23]. The aim of therapeutic cancer vaccines is to stimulate the patient’s adaptive immune system against specific tumor antigens to regain control over tumor growth, induce regression of established tumors and eradicate minimal residual disease [22,24].

The presence of PTMs in TAAs increases the immunogenicity as they may be considered foreign antigens by the immune system, or break the tolerance established against the self-unmodified protein (see Graphical Abstract). Immunogenic epitopes of TAAs elicit immune responses, especially the production of autoantibodies [25]. In general, TAA phosphorylated epitopes are better presented than non-phosphorylated epitopes by human leucocyte antigens (HLAs) [26,27,28].

Some key examples of PTM-modified TAAs and the relative induced immune responses are listed in Table 1. All the studies reporting specific T cells or antibodies against the modified TAA always analyzed the same response induced by the unmodified antigen.

Below, we will discuss in greater detail each PTM relevant for cancer immunotherapy.

## 2. Acetylation

**Figure 1:**
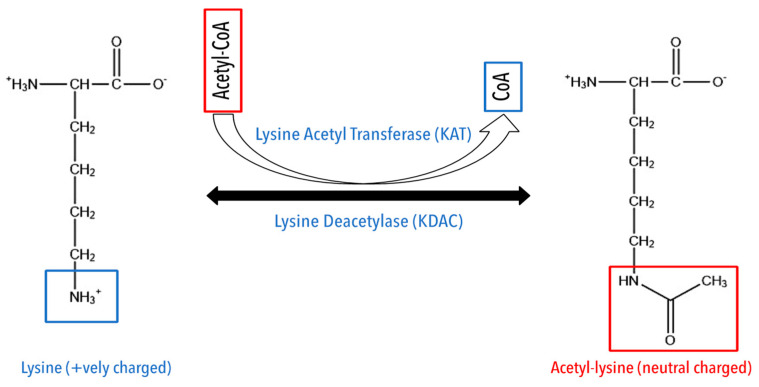
Schematic representation of the acetylation process.

Lysine (K) acetylation is a reversible PTM, which converts a positively charged lysine into a neutral amino acid, changing the function and properties of the protein. Acetylation plays an essential role in transcription regulation by modifying the core histone tails through lysine acetyltransferase (KAT) or lysine deacetylases (KDACs) (Figure 1) [45,46]. Acetylation in the histone protein is a crucial regulator in the transcription process [47,48]. Due to technical difficulties, acetylation was previously studied at the protein-to-protein level, which had restricted acetylation to be a nuclear PTM. With the advancement in enrichment methods and high-resolution mass spectrometry, studying acetylation at the proteome level became possible. These advanced methods led to identifying acetylation even in cytosolic and membrane proteins [49,50,51].

Altered acetylation levels in histone and non-histone proteins have been shown to play a role in tumorigenesis in numerous cancer types [52,53,54,55,56,57,58,59]. Gene expression of proto-oncogene gets activated during the hyperacetylation of histone. Histone acetylation is a reversible process for cancer therapeutics [60,61]. Five inhibitors of KDAC have been approved by the American Food and Drug Administration (FDA) for treating myeloma and T cell lymphoma [62]. The successful approval of five agents has pushed scientific communities to test the efficacy of KDAC inhibitors in other tumors. Many agents targeting KDAC have shown promising results in multiple clinical trials [61,63,64,65,66,67,68]. These results suggest that acetylation could represent a good target for tumor treatment, but none of these studies evaluated the potential presentation of acetylated histone epitopes or how the specific anti-tumor response was affected. Therefore, we did not include them in our discussion.

Besides histones, acetylation regards many other cytoplasmic proteins, such as the lactate dehydrogenase-A (LDH-A). In this case, acetylation inhibited its enzymatic activity, and acetylation of K5 in LDH-A in the pancreatic ductal adenocarcinoma (PDAC) cell line BxPC-3 was suggested to play a role in supporting tumor cell proliferation [69]. On the other hand, K5 acetylation of LDH-A also decreases lactate production thereby restraining pancreatic cancer cell migration. However, in human pancreatic cancer samples, a significant decrease in the ratio of K5 acetylated LDH-A to total LDH-A protein was observed, and acetylated LDH-A correlated with the tumor stage. This suggested a possible role of LDH-A-K5 acetylation in the initiation of pancreatic cancer but not in its progression [69]. Linear trap quadrupole-orbitrap mass spectrometry identified 26 acetylation sites in ENO1 from PDAC and normal pancreatic duct cells, and, of those, 5 were unique to PDAC cells [16]. ENO1 is a cytosolic or nuclear protein, expressed on the membrane wall of bacteria to help in their invasion [70]. In tumor cells, ENO1 is also highly expressed on the cell surface, but the mechanism by which it switches from cytoplasm to membrane is unknown [12]. It is supposed that PTM could represent one of the molecular mechanisms for its membrane exposure.

A pilot study to test whether or not acetylated peptides could be immunogenic was performed with p53 peptides. CD4 T cells were stimulated in vitro with autologous DCs pulsed with acetylated or non-acetylated p53 peptides [19]. A three-fold increased cytokine production was observed when CD4 T cells were stimulated with acetylated p53 peptides compared to non-acetylated p53 peptides. This T cell response was inhibited by the addition of an anti-HLA-DR but not by other anti-HLA class II (HLA-DQ and HLA-DP) antibodies, suggesting that the peptides (acetylated or non-acetylated) are mainly presented by HLA-DR molecules. Cancer patient peripheral blood mononuclear cells (PBMCs), but not healthy donor PBMCs, were able to specifically produce IFNγ after 7 days of stimulation with acetylated peptides but not with non-acetylated p53 peptides [19]. These results demonstrated that tumor-associated acetylated peptides are good candidates for developing cancer vaccines.

## 3. Citrullination

**Figure 2:**
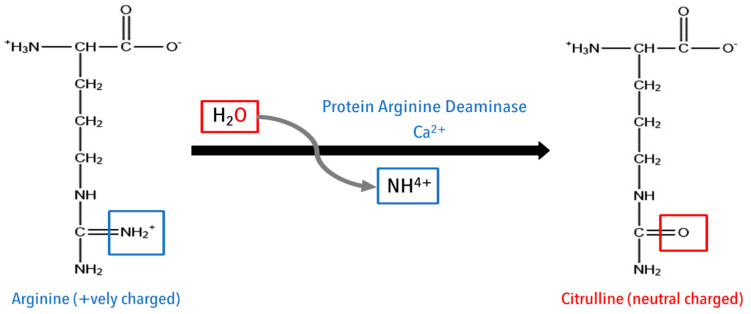
Schematic representation of citrullination.

Unlike other PTMs, citrullination is an irreversible modification, which converts the positively charged amino acid arginine (Arg) to neutral citrulline by a family of peptidyl arginine deiminase (PAD) enzymes [71]. The process is called citrullination or deamination (Figure 2); PAD replaces the primary ketamine (=NH) group in Arg with a ketone (=O) group, which was implicated in the recognition from the T cell receptor via HLA presentation [72,73]. The loss of positive charge affects the protein–protein interaction and the protein structure, and may lead to protein denaturation. Citrullination is a standard process observed in cells under stress, during nutrient starvation, and during apoptosis due to an increase in PAD expression [71].

A number of studies have reported that hypercitrullination could be a factor in breaking immune tolerance and inducing autoimmune diseases like type 1 diabetes, multiple sclerosis, and rheumatoid arthritis [4,72,73,74,75,76]. In rheumatoid arthritis, anti-cyclic citrullinated peptides in patient sera are biomarkers to identify the disease at early stages [4,75]. Given the role of citrullination in autoimmune diseases and its ability to break immune tolerance, the hypothesis that citrullinated peptides could also be immunotherapeutic agents in cancer treatments was evaluated.

Citrullinated peptides from Vim and ENO1 elicited a specific immune response with strong IFNγ release in HLA-DR4 transgenic mice, whereas there was no response against non-citrullinated peptides [34,37]. CD4 T cells were the most involved in mediating the citrullinated-specific response but displayed cytotoxic activity by expressing granzyme and Fas Ligand and directly killing tumors expressing HLA-II [34,37,77]. Splenocytes from mice immunized with citrullinated Vim peptides released granzyme upon stimulation with specific stimuli. Immunization with citrullinated [34,37,77] Vim and ENO1 peptides increase survival of HLA-DR4 transgenic mice implanted with B16F1 tumors expressing HLA-DR4, as well as Lewis lung carcinoma cells (LLC/2), ovarian cancer cells (ID8), and pancreatic cancer cells (Pan02) [17]. 

A proliferative response against citrullinated Vim and ENO1 peptides was observed in 67% of healthy donors of PBMC [36]. Only 28% of these healthy donors displayed HLA-DR4, whereas 71% of donors displayed HLA-DP4 [36]. By assessing the T cell repertoire to citrullinated peptides in ovarian cancer patients and healthy donors, it was demonstrated that PBMC from 58% of patients proliferated in response to at least one of the PTM peptides and only 12% to both citrullinated Vim and ENO1 peptides [17]. Analyzing the type of HLA revealed that most responders were HLA-DR4 or HLA-DP4, but not all. With predictive methodologies, it was found that some even expressed HLA-DQ6, HLA-DR13, and HLA-DP18 [17]. These data suggest that more HLA loci present citrullinated peptides, and that citrullinated peptides from TAA represent good candidates for vaccine approaches [17,77]. 

The binding of citrullinated Vim and ENO1 peptides to HLA-DP4 was tested by comparing to that of HLA-DP4-known binding peptides like those from the hepatitis B virus. Unmodified Vim aa415-423 and aa28-49 peptides showed low binding to HLA-DP4 compared to the citrullinated Vim peptides, which showed stronger binding. Similarly, citrullinated ENO1 peptide had higher binding capacity to HLA-DP4 compared to unmodified ENO1 peptide [37]. In HLA-DP4 transgenic mice, the combination of a vaccine composed of citrullinated Vim and ENO1 peptides with granulocyte–macrophage colony-stimulating factor (GM-CSF) and TLR agonists (especially with TLR1/2 agonist) reduced by 10- to 100-fold the dose of vaccine without losing the anti-tumor activity [40]. 

A combination of citrullinated peptides (Vim and ENO1) could also elicit similar immune responses in HLA-DR4 or HLA-DP4 transgenic mice [17,77]. These combination peptide vaccines could elicit anti-tumor therapy against multiple tumor models in mice [17,77].

In a B16 melanoma mouse model, citrullinated peptides induce IL10 release, but also higher secretion of IFNγ compared to non-citrullinated peptides [35]. New citrullinated peptides could be extracted by peptide elution and mass spectrometry [35]. Another important confirmation of the relevance of modified and specifically citrullinated peptides as targets to elicit an anti-tumor response is the presence of elevated levels of IgG bound citrullinated peptides in the sera of newly diagnosed breast cancer patients (0–0.8 years) [78]. This suggests that citrullinated peptides-Ig complexes could be explored as biomarkers for early detection just as they are used in the early identification of RA.

## 4. Phosphorylation

**Figure 3:**
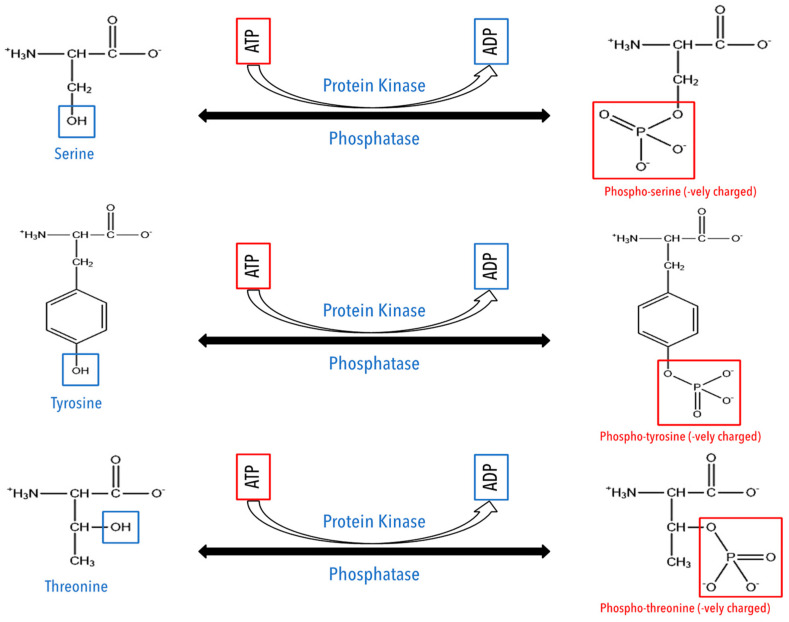
Schematic representation of phosphorylation on multiple amino acids.

Phosphorylation is a reversible PTM catalyzed by phosphotransferase, which adds a phosphate group on the hydroxyl group of amino acid residues (Ser/Thr/Tyr) from the ATP molecule (Figure 3) [79]. It is one of the most widely studied PTMs [2]. One of the hallmarks of tumor growth is, in fact, dysregulated phosphorylation, which contributes directly to oncogenic signaling cascades involved in cell growth, differentiation, and survival [80,81,82,83]. This renders phosphorylation an interesting potential therapeutic tool, as the presence of PTMs increases the variety of naturally occurring peptide epitopes [84,85,86].

Phosphorylated peptides can be presented by HLA-II molecules. Structural analysis showed a 2.1 Å resolution of phosphorylated tumor-associated antigen MART-1 peptide (pMART-1_100–114_) bound with HLA-DR1 [27]. Specific CD4^+^ T cell clones secreted GM-CSF in response to phosphorylated MART-1 peptide pulsed onto the HLA-DR1-expressing antigen presenting cells (APCs), but not to the non-phosphorylated peptide. This demonstrates that the phosphate group is indeed a critical determinant for T cell receptor recognition [27].

A study validated the hypothesis that phosphopeptides can be immunotherapeutic targets by analyzing mixtures of more than 10,000 peptides presented by HLA-A*201 on the surface of human melanoma, ovarian cancer, and B cell lymphoma cell lines [31]. They were isolated and extracted, and phosphopeptides were enriched; 36 phosphopeptides presented by HLA-A*201 on one or more of the four cell lines were identified, sequenced, and employed to immunize transgenic mice expressing HLA-A*201. Isolated phosphopeptide-specific CD8 T cells secreted IFNγ when exposed to synthetic phosphopeptide epitopes, but not to non-phosphorylated peptides [31]. Fresh PBMC from melanoma patients HLA-DRB1*01, HLA-DQB1*0501 were stimulated in vitro with phosphorylated MART1 (pMART1) peptides for several rounds of simulation. Phosphorylated specific CD4+ T cells secreted IFNγ and GM-CSF in response to pMART1 but not in response to non-phosphorylated MART1 [29].

A phosphorylation site (Ser419) was also identified in the more acidic isoforms of the glycolytic enzyme ENO1 in PDAC and normal pancreatic ductal cells [16]. Autoantibodies against phosphorylated ENO1 were found in a greater percentage of PDAC patients, and only in a small percentage of healthy individuals [14]. Antibodies present in the sera of PDA patients recognized six different isoforms of ENO1 (ENO_1,2,3,4,5,6) while those in healthy individuals recognized only four isoforms. Notably, the presence of anti-ENO_1,2 autoantibodies improved the diagnostic performance of CA19.9 in pancreatic cancer patients with low levels of CA19.9, and correlated with a better prognosis and overall survival (OS) [14]. This suggests that phosphorylation plays a role in breaking tolerance in cancer patients in the attempt to fight tumor growth. The association between the presence of the HLA-DRB1*08 allele and the production of autoantibodies against a phosphorylated epitope of ENO1 was also demonstrated [15]. In effect, HLA-DRB1*08 allele was more frequent in PDAC patients with autoantibodies against pENO1_413-422_ (phosphate group at Ser419) than healthy controls or patients without these autoantibodies. Interestingly, PDAC patients with autoantibodies against pENO1 also displayed T cells that proliferated and secreted IFNγ in response to phosphopeptides, but to a lesser extent in response to unmodified peptides [15].

Phosphopeptides from IRS2, CDC25b, p53, Vim, and TRAP1 also could elicit specific immune responses in different cancer models in terms of T cells secreting IFNγ [21,31,32,33]. Phosphopeptides of IRS-2, CDC25b, and TRAP1 specifically elicited CD8 T cells, whereas the CD4 specific T cell response was elicited in response to phosphopeptides of p53 and Vim [21,31,32,33].

An open-label, pilot, proof-of-concept clinical trial study to assess the phosphopeptide vaccine safety and immunogenicity was performed on patients with resected stage II–IV melanoma [30,87]. pIRS2 and phosphorylated BCAR3 were used as vaccines. Patients were divided into three groups: the first group (three patients) was administered with pBCAR3, the second group (three patients) with pIRS2, and the third group (nine patients) received both phosphopeptides. Vaccines were administered along with tetanus toxoid peptide in a water-in-oil emulsion with an equal volume of incomplete Freud’s adjuvant. Immediately after vaccination, poly-L-lysine and carboxymethyl cellulose were injected in patients to stimulate the immune system [30]. A total of 17% of patients administered with pBCAR3 showed a CD8+ T cell response, whereas 42% of patients elicited a CD8+ T cell response when administered with the pIRS2 vaccine, with a greater increase in IFNγ production [30,87]. None of the patients reported severe adverse effects after vaccination, showing that the phosphopeptide vaccine is safe and should be tried in larger clinical trials [30,87]. 

Overall, all these studies support the hypothesis that phosphorylated epitopes are recognized by the adaptive immune system and, therefore, imply that the antitumor response can fight tumor growth.

## 5. Glycosylation

Protein glycosylation is a PTM where a carbohydrate molecule is attached to nitrogen or hydroxyl or other functional groups of amino acids through enzymatic reactions. Glycosyltransferase enzyme catalyzes these reactions [88]. Protein glycosylation is classified into two major categories: N-Linked glycosylation, where glycans are attached to the nitrogen of an Asparagine (Asn) or Arg residues, and O-Linked glycosylation, where glycans are attached to the hydroxyl group of Ser or Thr residues [88]. Protein glycosylation plays a significant role in protein folding, activity, stability, and conformation. Almost half of human proteins are glycosylated, and the majority of cancer biomarkers, which have been approved by FDA, consist of glycoprotein or carbohydrate antigens [89,90,91,92]. 

Many glycoproteins are associated with cancer progression [42,93]. One common glycoprotein whose role is well established in tumors is MUC. The first identified membrane MUC protein in many solid tumors and hematopoietic cancers was MUC1 [39,94,95]. MUC1 is often upregulated and aberrantly glycosylated, making it a potential therapeutic target for cancer immunotherapy. In some malignant transformations, MUC1 becomes hypo-glycosylated carrying truncated carbohydrates known as Tn antigens. MUC2 is another MUC protein, commonly found in intestinal lining and expressed in goblet cells of the small bowel and colon [96]. In mucinous carcinoma of the pancreas, prostate, breast, ovary, and colon, there is an overexpression of MUC2 [97,98].

The potential of MUC1 as a vaccine was evaluated in pre-clinical models in different tumors [41,43,93,99,100,101,102]. Successful results in mice led to many phase I clinical trials showing that MUC1 is safe and well tolerated in patients [20,103,104,105,106,107,108,109,110]. These exciting results began a new era of immunotherapy clinical trials over the following two decades. Many phase II and phase III clinical trials targeting glycosylation demonstrated an effective antitumor response, but limited success in extending survival of cancer patients. [111,112,113,114,115,116,117,118,119,120,121] Phase III clinical trials targeting MUC1 are shown in Table 2.

The majority of clinical trials proposed the use of a viral vector expressing MUC1 alone or in combination with other TAA in different solid cancers [105,109,114,115,118,121,122,123,124,125]. Many phase III trials got terminated either prematurely or suspended because of lack of funding; hence, there are no related publications and limited information in the Clinicaltrials.gov website (access on 20 December 2022). One of the major phase III clinical trials used TG4010, a modified vaccinia Ankara vector expressing MUC1 and interleukin-2, in combination with chemotherapeutic drugs or placebo in 222 non-small cell lung cancer (NSCLC) patients [114]. Patients were subdivided into two arms: those receiving chemotherapeutic drugs with TG4010 and those receiving chemotherapeutic medication with a placebo. Patients treated with TG4010 combined with chemotherapeutic drugs had a longer significant PFS compared to that of patients treated with a placebo plus chemotherapeutic drugs [114].

The most extensive phase III clinical trial of MUC1 was conducted by enrolling 1513 patients of NSCLC treated with tecemotide, a lipopeptide derived from MUC1 [120]. No significant differences in OS of the patients treated with tecemotide or with placebo were reported; however, 10.2 months improvement in median survival for patients who received tecemotide after chemoradiotherapy was observed. This suggest that tecemotide may have a potential role in the efficacy of maintenance therapy after initial concurrent chemoradiotherapy in NSCLC patients [120]. Other phase III clinical trials targeting MUC1 are detailed in Table 2 with outcomes from the studies.

Many small Phase I/II trials evaluated the potential of autologous DC presenting MUC1 [107,108,110,119,126,127]. The vaccine (CVac) was made with a recombinant fusion protein (FP) conjugated to oxidized mannan (M) and loaded into autologous DC [107]. The FP consisted of a variable number of tandem repeats (VNTR) region of the MUC1 protein and glutathione-S-transferase [107]. The benefit of mannan-conjugated antigen is to ability to induce DC activation and maturation by targeting the complex of mannose receptor of DC [107]. Oxidized mannan increases the efficiency of the HLA I presentation of MUC1 recombinant protein [128]. Ovarian cancer patients in complete remission (CR) were treated with CVac to evaluate its efficacy and safety [127]. Patients from first CR (CR1) or second CR (CR2) were randomised to standard of care (SOC) or CVac-treatment. Patients were given 10 doses of CVac over 56 weeks and followed-up for another 48 weeks at the end of the study to measure PFS. When both groups were challenged with MUC1 antigen, SOC patients had few or no T cells, whereas CVac treated patients had both CD8 and CD4 T cell responses [127]. In addition, CVac-treated patients displayed a higher CD8 cytotoxic T cell response compared to that elicited in CD4 T cells, for which it was not possible to evaluate the release of cytokines. In the CR1 group of patients no significant change was observed in progression-free survival (PFS) and OS between the SOC and CVac arms. However in CR2 patients, CVac-treated patients had higher PFS compared with the SOC control group [127]. Another phase I/II clinical trial assessed the safety of a vaccine composed of Tn-MUC1 loaded onto autologous DC in 17 patients with non-metastatic castrate resistant prostate cancer (nmCRPC) [126]. The Tn-MUC1 DC vaccine was found to be safe, and elicited a strong CD4 T cell response by increasing the secretion of cytokines such as TNFα, IL2, IFNγ, and a more robust CD8 T cell response, although nmCRPC patients did not achieve the desired prostate-specific antigen (PSA) values [126]. However, the strong immune response monitored in the patients suggested that a larger trial in combination with chemotherapeutic drugs could improve both PFS and OS. 

Many clinical trials also employed direct vaccination with MUC1 peptides in different tumors, and even as a preventing strategy [20,104,106,129,130]. Patients with advanced colon adenoma were vaccinated with MUC1 to assess the ability of this vaccine to induce an anti-MUC1 immune response and long-term memory without toxicity [129]. The authors found that 44% of patients were able to produce high levels of anti-MUC1 immunoglobulin G (IgG) and long-lasting immune memory without any toxicity [129]. The remaining 56% of patients, who did not show elevated levels of anti-MUC1 IgG, displayed higher levels of myeloid derived suppressor cells (MSDC) already before the vaccination [129]. Another interesting phase I study used a humanized glycol-optimized monoclonal antibody against the MUC1 epitope (PankoMab-GEX) in different cancers [130]. PankoMab-GEX was well tolerated, and in patients with advanced disease was strong enough to elicit an anti-tumor activity. The best result in this trial was observed in ovarian and lung cancer patients: in the former cohort, one patient had a complete response, and 32% of patients displayed disease stabilization [130]. 

MUC2 is commonly used as a biomarker for many cancers as well as other diseases [131,132,133,134]. A study with fifty patients with goblet cell metaplasia found that MUC2 expression in non-goblet epithelium may represent a specific biomarker. The authors concluded that, in the esophagus, MUC2 expression represents a late event in the conversion of mucinous columnar cells to goblet cells [134]. MUC2 overexpression was correlated with the lower tumor grade and lower rate of lymphatic invasion in a large meta-analysis of 2363 patients of gastric carcinoma [135]. However, there was no statistically significant correlation between the expression of MUC2 and lymph node metastasis, gender, and five-year survival rate [135].

Clinical studies assessed the role of MUC2 as a potential therapeutic immune target in cancers [136,137,138,139,140]. MUC2, conjugated to the immunologic carrier protein, keyhole limpet hemocyanin (KLH), and given with the saponin adjuvant, *Quillaja saponin* (QS-21) was safe and induced high IgM and IgG titers specific for the immunogen [139,140,141,142]. Another interesting tumor-associated carbohydrate antigen is Globo H, which is expressed on the outer membrane of cancer cells but not in normal tissue cells. Indeed, antibodies against Globo H mediated complement lysis or ADCC [141]. In a phase I clinical trial, a bivalent vaccine consisting of Globo H and MUC2 conjugated to the carrier, KLH, and mixed with adjuvant QS21 was administered to 43 patients with relapsed prostate cancer [143]. The vaccine was found to be safe and generated high titer of IgG and IgM antibodies to MUC2, but only IgM antibodies to Globo-H [143]. The promising result from Phase I clinical trial led to other Phase II clinical trials involving MUC2 and/or Globo H as vaccine targets with conjugates, which have no posted results yet [NCT00036933; NCT00004929; NCT00016146].

Even after some failed trials, an interest in finding a vaccine for cancer using glycosylated antigens as a target has not decreased, and the number of new registrations for clinical trials have increased in the past two decades. This clearly shows the faith of corporations in investing a lot in immunotherapy.

## 6. Conclusions

It is becoming clearer that immunotherapy represents real promise for treating cancer, in all its forms. Passive immunization and immune checkpoint blockade were the first approaches to take hold, although an increase in clinical trials using the adoptive transfer of CAR T cells or TRC-engineered T cells has been reported since 2015 [145]. Vaccines are still less successful compared to previous approaches due to the complex relationship between tumor, stroma, and immune cells, which render the microenvironment extremely demanding and exhausting. However, the crucial point in the active immunotherapy remains the choice of antigen to be targeted by the vaccine. TAAs with PTMs represent an interesting option for cancer immunotherapy. New insights regarding the ability of acetylated and citrullinated peptides to elicit tumor specific responses represent promising results that, nevertheless, need further investigation, especially in pilot phase clinical trials. The promising results from combinational citrullinated ENO1 and Vim peptides also should be further explored with chemotherapeutic targets in pilot clinical trials for their immunogenicity and safety in cancer patients, to open new possibilities of design immunotherapeutic strategies. Efforts should first be focused on identifying, and then exploiting, aberrant phosphorylated, acetylated, citrullinated, and glycosylated proteins variably expressed from multiple cancers to develop vaccines for large scale immunotherapy. The focus should be to translate these preclinical studies into clinical trials. 

A pilot clinical trial into vaccines against phosphorylated peptides of pIRS2 and PBCAR3 along with the vaccine against MUC1 and MUC2 showed promising results for those many vaccines which have yet to undergo clinical trials. Future vaccine strategies could involve many PTM antigens, which could enhance the magnitude of the immune response. 

It is very important to deeply understand the meaning of PTMs in cancer in parallel to their immunogenicity characterization. If the PTM becomes a general and key process acquired during carcinogenesis, it is expected that it will be maintained in all tumor cells and not only in certain clones, which happens for the so-called neoantigens. This will allow the extreme heterogeneity that has been well described in tumor cells to be overcome. Are PTMs modulated by treatments, and, if yes, in which way and with which drugs? The answer to this open question will also allow designing the best vaccine for each patient based on which treatment they are receiving.

## Figures and Tables

**Table 1:** Immune response elicited by PTM-modified TAA.

PTMs	TAA	Type of Tumor in Which TAA Has Been Identified or to Which the Antigen Is Associated	Immune Recognition (If Any)	References
**Acetylation**	ENO1	Pancreas	NA	[16]
	p53	Colon, Prostrate, Pharynx	CD4+ T Cell	[19]
**Phosphorylation**	MART-1	Melanoma, Leukemia	CD4+ T Cell	[29]
	IRS2	Melanoma, Breast, Ovary, Colon	CD8+ T Cell	[18,30,31]
	β-Catenin	Ovary, Melanoma	CD8+ T Cell	[30,31]
	Breast cancer antiestrogen resistance 3	Melanoma	CD8+ T Cell	[30]
	p53	Head and Neck	CD4+ T Cell	[21]
	ENO1	Pancreas	Ab, CD4+ T Cell	[14,15]
	CDC25b	Melanoma, Breast, Ovary, Colon, Leukemia	CD8+ T Cell	[18,31]
	TNF receptor associated protein (TRAP-1)	Lung	CD8+ T Cell	[32]
	Vim	Colon	CD4+ T cells	[33]
**Citrullination**	Vim	Melanoma, Lung	CD4+ T Cell	[17,34,35,36]
	Enolase	Melanoma, Lung	CD4+ T Cell	[17,35,36,37]
**Glycosylation**	MUC1	Breast, Ovary	CD8+ T Cell, Ab	[38,39]
**Sialylation**	Silayl-Tn-Antigen	Breast, Ovary	Ab (IgM)	[40,41,42,43]
**SUMOylation**	p53	Sarcoma	NA	[44]
**Methylation**	Enolase	Pancreas	NA	[16]

**Table 2:** List of Phase III clinical trials using MUC1 antigen (retrieved from clinicaltrial.gov website, accessed on 9 September 2022).

Vaccine	Number of Patients	Treatment	Outcome	References
Oxidized mannan MUC1 peptide	31 doubly blind breast cancer stage II	Administered subcutaneous injections of either placebo or oxidized mannan- MUC1	5.5 years since the final patient began treatment (8.5 years from the start of treatment of the first patient); the recurrence rate in patients receiving the placebo was 27% (4/15; the expected rate of recurrence in stage II breast cancer); those receiving immunotherapy had no recurrences (0/16); and this finding was statistically significant (*P* = 0.0292).	[112]
PANVAC-VF viral vector expressing CEA. and MUC1 plus B7.1,	255 advanced pancreatic cancer patients	PANVAC-VF versus palliative chemotherapy	No significant difference in OS of patients receiving PANVAC-VF versus palliative chemotherapy or best supportive care	[144]
Silayl Tn-KLH	1028 breast cancer patients	Silayl Tn-KLH versus KLH	No significant difference in OS in patients receiving Silayl Tn-KLH versus KLH alone	[117]
Tecemotide (L-BLP25) lyophilized 25mer MUC1	1513 NSCLC patients	Tecemotide (L-BLP25) versus placebo after chemoradiotherapy	No significant OS difference within whole cohort	[120]
TG4010 (a modified vaccinia Ankara expressing MUC1) and interleukin 2	222 stage IV NSCLC patients (phase 2b/3)		TG4010 plus chemotherapy seems to improve progression-free survival compared to placebo plus chemotherapy	[114]
Tecemotide (L-BLP25) lyophilized 25mer MUC1	285 Stage IV NSCLC patients		Study was prematurely terminated	[111,116]

OS: overall survival; PANVAC-VF: cancer vaccine targeting MUC1, and carcinoembryonic antigen delivered via two viral vector vaccina (V) and flowpox (F); KLH: keyhole limpet hemocyanin.
